# The bioengineered HALOA complex induces anoikis in chronic myeloid leukemia cells by targeting the BCR-ABL/Notch/Ikaros/Redox/Inflammation axis

**DOI:** 10.25122/jml-2021-0230

**Published:** 2022-05

**Authors:** Vivek Singh, Ranjana Singh, Abbas Ali Mahdi, Anil Kumar Tripathi

**Affiliations:** 1. Department of Biochemistry, King George's Medical University, Lucknow, Uttar Pradesh, India; 2. Department of Clinical Hematology, King George's Medical University, Lucknow, Uttar Pradesh, India

**Keywords:** BCR-ABL Translocation, ROS – Reactive oxygen species, RNS – Reactive nitrogen species, Leptin, IL-8, MMP-9, CCAR1, Notch signaling pathway, BC – Blast crisis, CML – Chronic myeloid leukemia, HALOA complex – Human alpha-lactalbumin oleic acid.

## Abstract

Blast crisis (BC) is an outcome that arises during the treatment process of chronic myeloid leukemia (CML), which is possibly attained by the dysregulation of the Notch and Ikaros signaling pathways, BCR-ABL translocation, redox, and inflammatory factors. This study demonstrated that biotherapeutic agents target aberrant molecular axis in CML-BC cells. The HALOA complex was synthesized by simple mixing of apo α-lactalbumin with oleic acid, which manages to inhibit BCR-ABL (b3a2 in K562 cells) translocation. It elevates the production of reactive oxygen species (ROS), reactive nitrogen species (RNS), and protein carbonyl, which induces DNA fragmentation in K562 cells but not in NIH cells. The complex manages to reduce the toxicity surrounding apoptotic cells by enhancing the production of superoxide dismutase (SOD) and the total antioxidant level. The HALOA complex increases leptin to maintain normoxic conditions, ultimately preventing angiogenesis. This complex downregulates the expression of IL-8 and MMP-9 and elevates the expression levels of Notch 4, Ikaros, and integrin alpha-D/CD-11d (tumor-suppressive), which conjointly prevents inflammation, metastasis, and epithelial-mesenchymal transition (EMT) in CML cells. Meanwhile, the complex downregulates Notch 1 and 2 (oncogenic), consequently inducing anoikis in CML cells. Overall, the HALOA complex shows credibility by targeting the combined molecular factors responsible for the pathogenesis of the disease and will also help to overcome MDR conditions in leukemia.

## INTRODUCTION

Chronic myeloid leukemia (CML) is a myeloproliferative disorder distinguished by the Philadelphia (Ph) chromosome [1]. The molecular consequences of the Ph chromosome form the BCR-ABL oncogene that encodes a chimeric BCR-ABL oncoprotein, which disrupts several factors (leptin, IL-8, MMP-9, and survivin) and pathways (Ikaros/Notch/CD-11d signaling pathways) responsible for the pathogenesis of leukemia. Several reports documented that BCR-ABL knockdown in the K562 cell line inhibits the notch signaling pathways, and this inhibition shows that notch (1 and 2) have an oncogenic role in CML [2]. In the transgenic CML mouse model, the overexpression of BCR-ABL led to the activation of notch-1. Several studies have suggested that in K562 cells, notch-1 is continuously expressed after treatment with gamma-secretase inhibitor (GSI) [3]. However, Ikaros was downregulated in BCR-ABL-positive CML cells in blast crisis [4], which transcriptionally regulates the Notch 1 protein during normal hematopoiesis. Previous literature has described that notch-1 and 2 act as oncogenic agents, while notch-4 functions as a tumor suppressor [5, 6].

Moreover, Notch-4 downregulates epithelial-mesenchymal transition (EMT) in cancer cells, as indicated by a marker (MMP-9). MMP-9 is characterized as a gelatinase that preferentially degrades intact collagen, which leads to oncogenesis mediated by various oncogenic factors, such as vascular endothelial growth factor, fibroblast growth factor, and IL-8 [7]. Furthermore, the treatment of endothelial cells with IL-8 significantly enhances the production of MMP-9, which downregulates leptin, leading to the formation of capillary tube organization (angiogenesis). MMP-9 also negatively regulates CD11d in BCR-ABL-positive cells, which promotes angiogenesis. However, CD11d (integrin alpha-D/ITGAD) facilitates the clearance of apoptotic cells. Thus, IL-8 might be used as a biomarker for tracking CML inhibitor efficacy and can play a potential role in the pathophysiology of CML. However, IL-8 represents one of the most stable and potent families of neutrophil chemotactic and activating factors, which is induced by oxidative stress [8, 9], and antioxidants inhibit IL-8 expression [10, 11]. In this context, the objective of this study was to assess the therapeutic efficacy of the HALOA complex on K562 cell lines and their effect on the redox (oxidative stress and antioxidant capacity) system. Several factors, such as survivin, IL-8, leptin, and MMP-9, are responsible for the pathogenesis of CML. Increasing evidence points towards GSI/TKI showing no efficacy as an anticancer agent in BCR-ABL leukemia cells. In this study, we checked the therapeutic efficacy of the complex on CML cells by targeting the BCR-ABL and Notch signaling pathways (Notch-1, 2, and 4) and the Ikaros protein.

## MATERIAL AND METHODS

### HALOA complex formation

We have previously formulated and characterized the HALOA complex [12].

### Cell culture

K562 (human chronic myeloid leukemia cells) and NIH (fibroblast cells) cells were purchased from the National Centre for Cell Science (India). NIH cells were cultured in Dulbecco's modified Eagle's medium (DMEM) with 2 mM L-glutamine and supplemented with 10% fetal bovine serum, antimycotic and antibacterial (0.1%) until 90–95% confluence before being subcultivated. K562 cells were maintained in a culture medium composed of RPMI-1640, fetal bovine serum (5%), antimycotic, and antibacterial (0.1%). During experiments, 1 × 10^6^ cells were seeded in 150 mm diameter dishes. The K562 and NIH-3T3 cells were maintained in a humidified incubator at 37°C with 5% CO2. Before the treatment, the cells were examined for mycoplasma contamination.

### MTT assay for cell viability

To determine possible cytotoxic effects induced by the HALOA complex, 3-(4,5-dimethylthiazol-2-yl)-2,5-diphenyltetrazolium bromide (MTT Abcam assay) was used. K562 cells were seeded into 96-well plates (Thermo Scientific) (10000 cells per well). The cells were treated for 24, 48 and 72 hours with different concentrations of HALOA: 1 mg/mL, 0.5 mg/mL, 0.25 mg/mL, 0.125 mg/mL, Apo α-LA; 1 mg/mL, and OA; 1 mg/mL 24 hours after seeding. Untreated K562 cells as controls were also incubated for 24, 48, and 72 hours. After incubation, the cells were washed with PBS and treated with 20 µL MTT solution per well for 2 hours at 37°C. Subsequently, the supernatants were removed, and 50 µL dimethyl sulfoxide (DMSO Thermo Scientific) was added. To dissolve the formazan salt, the plates were centrifuged at 5000 rpm for 5 minutes at room temperature. The absorbance at 540 nm was recorded using a microplate reader (Thermo Scientific Multiscan-Go) [12].

### BCR-ABL translocation by multiplex PCR

A multiplex RT-PCR assay was performed with a Seeplex leukemia BCR/ABL kit (Seegene, Seoul, Korea), The PCR products were analyzed with 2% agarose gel electrophoresis at 100 V for 60 minutes [13].

### Redox Assay

ROS/SOD by Abcam-ab139476 kit, Nitric oxide assay kit (colorimetric)-ab65328, protein carbonyl content assay kit-ab126287, Antioxidant assay kit-Sigma Aldrich; CS0790. All protocols were performed according to the manufacturer's instructions.

### Agarose gel electrophoresis analysis of DNA fragmentation

DNA fragmentation (DNA fragmentation kit-TaKaRa; 6137) was detected by agarose gel electrophoresis. The cell suspension remaining after Trypan blue (970 µL, 2 × 10^6^/mL) was lysed in 5 mM Tris, 20 mM EDTA, and 0.5% Triton X-100 (pH 8.0) at 4°C for 1 hour and centrifuged at 13,000 rpm for 15 minutes. DNA was ethanol precipitated overnight at -20°C, further treated with proteinase K and RNAse, loaded on 1.0% agarose gels, and electrophoresis maintained at a constant voltage of 70 V for 2 hours. Fragmentation of DNA was visualized with ethidium bromide using the Bio-Rad gel doc.

### 18s rRNA analysis

TaqMan Fast Reagent Starter Kit (Applied Biosystems; 4352407). The reaction conditions were as follows: hold (20 seconds, 95°C), melt (1 second, 95°C), and annealing/extension (20 seconds, 95°C) for 40 cycles. Gel electrophoresis (2.5%) image taken from Bio-Rad gel doc.

### Inflammatory markers evaluation by ELISA assay

Leptin human simple step ELISA Kit (ab179884), Human IL-8 ELISA Kit (ab46032), Ray Bio Human MMP-9 ELISA Kit (ELH-MMP-9), Survivin Human ELISA Kit (ab119607).

### Gene expression by RT-PCR

Total RNA from cells was isolated by following the TRIzol method. The RNA concentration and structural integrity were confirmed using a NanoDrop 2000 UV–Vis spectrophotometer (Thermo Scientific). Only RNA with the ratios from 1.9–2.0 of absorbance at 260/280 nm was used. The isolated mRNA was reverse-transcribed using the High-Capacity c-DNA Reverse Transcription Kit (4368814) according to the protocol. Quantitative reverse transcription-PCR (q RT-PCR) was performed by using PowerUp SYBR green master mix (ABI-A25741) on a 7500 Fast Real-Time PCR system (Applied Biosystem, Thermo Scientific). Quantification was performed with the ΔΔ Ct method with β-actin serving as a reference gene, and the RT-PCR results were analyzed by DataAssist software (Thermo Scientific). The oligonucleotide primers are listed in Table S1. All primers were analyzed with positive controls by performing melting profiles following q RT-PCR, and product sizes were checked by 2.2% agarose gel electrophoresis. PCR conditions were as follows: 42 cycles of 15 seconds at 95°C, 15 seconds at annealing temperature (60°C for all other genes), and 15 seconds at 72°C. Specimens were assayed in duplicate for at least three independent experiments as indicated [14].

### Establishing the protein-protein interaction by the STRING web tool

Protein-protein interaction network functional enrichment analysis was performed using all the proteins from UniProtKB. (Results available on this server https://string-db.org/cgi/network.pl?taskId=PtDZgNYDNqgd) .

### The efficiency of the HALOA complex on Notch confirmed by the docking method

Using HDOCK software, where the input is Notch (PBD-4J2X), RBP.jk (PDB-2F8X) and human α-lactalbumin (PDB-1A4V) performed the docking.

### Statistical analysis

All the data are presented as the means±SD and analyzed using SPSS 17.0 and GraphPad software (Prism V).

## RESULTS

### Human alpha-lactalbumin oleic acid complex formation

We used human alpha-lactalbumin and oleic acid as the starting material and formulated the HALOA complex previously generated by our team, as shown in Figure S1 A. Furthermore, its antitumor activity on K562 and NIH cell lines was elucidated.

### Cell culture and cell viability assay

NIH (normal cell line to check toxicity of the complex) and K562 cells (chronic myeloid leukemia) were treated using different concentrations of HALOA: 1 mg/mL, 0.5 mg/mL, 0.25 mg/mL, 0.125 mg/mL, HLALB/Apo α-LA: 1 mg/mL, and OA: 1 mg/mL. Cell viability was determined at 24, 48, and 72 hours of treatment by adding an MTT reagent following the manufacturer's protocol. Then, we calculated the IC50, which indicates that 0.5 mg/mL complex shows the best result without any cell cytotoxicity, as shown in Figure S1 B, C. In addition, longer-term *in vitro* assays were performed, with viability assessed after suspension by Trypan blue.

### Complex inhibits the BCR-ABL translocation in CML cells

The standard bands (M) were as follows: -1012 bp (c3a2), 764 bp (b1a1), 600 bp (internal control), 476 bp (b3a2), 401 bp (b2a2), 348 bp (e1a2), 299 (b3a3), 224 bp (b2a3), and 174 bp (e2a3). The quality of RNA and the efficiency of cDNA synthesis were analyzed by amplification of the BCR gene as an internal control. In our study, the amplified product (600 bp) from the BCR gene was the only band detected in HALOA-treated K562 cells. The results of multiplex RT-PCR for each sample (PTC, K562, HALOA, HLALB, OA1, OA2, OA3) are shown in Figure 1 A, and the translocation results are shown in Table S2. The primer combinations in multiplex RT-PCR allowed simultaneous detection of all known types of BCR-ABL and BCR transcription in a single reaction. Using the SeeplexR leukemia BCR-ABL kit, we examined K562 cells and treated cells with the HALOA complex, HLALB, and OA (1,2,3) and found that HALOA complex-treated cells effectively inhibited all types of BCR-ABL translocation in comparison with their individual entities. K562 cells express p210 (b3a2) BCR-ABL translocation. In OA-treated cells, we found coexpression of fused translocation. However, HLALB itself downregulates the expression of b3a2 translocation (as reported in the majority of patients) in comparison with untreated K562 cells. Lane and band analyses were performed by Image Lab software, as shown in Figure 1 A (Figure S1 D–G). The detection limit of the Seeplex® Leukemia BCR/ABL was 10 copies/reaction (10 copies/3 µL nucleic acid). Reproducibility tests were carried out at 3 different time points in 2 weeks by 3 different experimenters. The same results were obtained in every test, confirming the reproducibility of the product.

**Figure 1:**
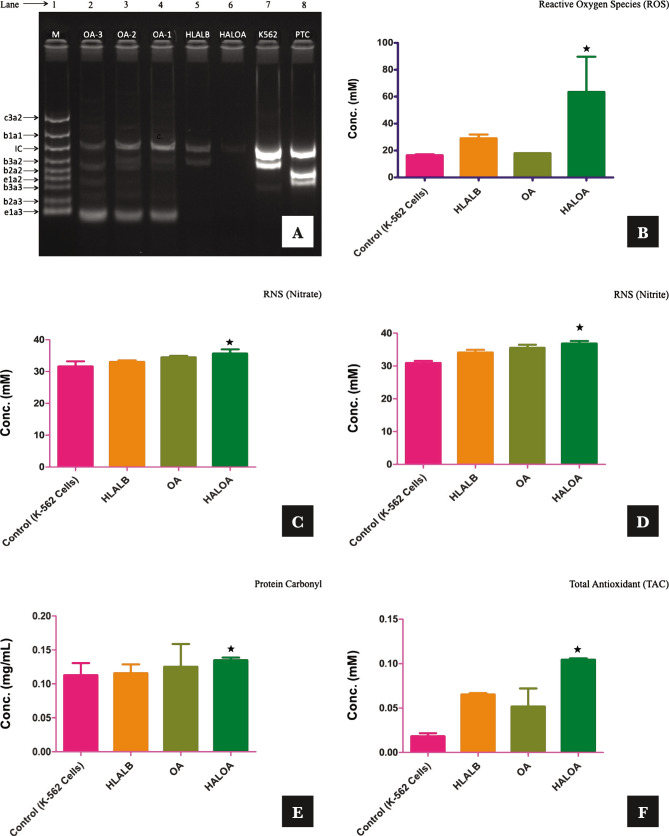
Overall validation of the HALOA complex as an antitumor agent on CML cells: (A) M indicated the 1012 bp (c3a2), 764 bp (b1a1), 600 bp (internal control), 476 bp (b3a2), 401bp (b2a2), 348 bp (e1a2), 299 (b3a3), 224 bp (b2a3), 174 bp (e2a3), PTC; b2a2, e1a2 translocation, K562; b3a2 (major), HALOA (Human alpha-lactalbumin oleic acid complex); No BCR-ABL translocation, HLALB (Human alpha-lactalbumin); b3a2 (down-regulated), OA1, 2, and 3 (Oleic, linoleic, linoic acid); e1a3 and fused translocation. (B) ROS (mM); Control- 16.5±0.7, HALOA- 63.5±26.1, p-value; 0.008. (C, D) RNS (Nitrate, Nitrite- mM); Control- 32.0±0.95, 30.9±0.63, HALOA- 35.6±1.13, p-value; 0.014, 36.8±0.77, p-value; 0.014. (E) Protein Carbonyl (mg/mL) conc. in control- 0.11±0.01, HALOA- 0.13±0.004, p-value; 0.0001. (F) TAC (mM); Control- 0.018±0.003, HALOA- 0.10±0.001, p-value; 0.01. (G) SOD (mM); Control- 1.0±0.0, HALOA- 4.5±0.70, p-value; 0.07. The results are shown as the mean±SD, and the graph shows the p-value (*<0.05). Please refer to supplementary Figure S1.

**Figure 1:**
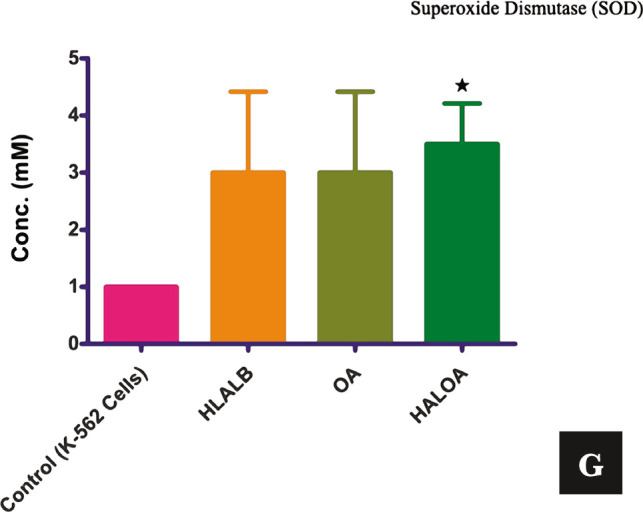
Continued.

### Effect of the complex on the redox system [oxidative (ROS, RNS, protein carbonyl) and antioxidative (TAC and SOD)] of K562 cells

Several reports illustrated that maximum drugs induce apoptosis in BCR-ABL-positive cells by enhancing the level of intracellular oxidative stress (ROS/RNS/protein carbonyl), which consequently leads to DNA fragmentation in CML cells [15].

We also explored the molecular action of the HALOA complex on redox biology in the K562 cell line (BCR-ABL positive) along with a normal cell line (NIH cells) and reported that the complex does not have any effect on NIH cells, showing no apoptosis. This study explored not only the ability of the complex to elevate the level of ROS/RNS/protein carbonyl but also the ability to enhance the total antioxidant capacity (TAC and SOD) and inhibit BCR-ABL translocation in K562 cells, as explained in Figure 1 A. We found that HALOA complex manage to elevate the level of ROS by 63.5±26.1 mM (p=0.008), RNS (Nitrate and Nitrite)- 35.6±1.13 mM (p=0.014), 36.8±0.77 mM (p=0.01), and Protein carbonyl- 0.13±0.004 mg/mL (p=0.001) in comparison with untreated K562 cell ROS- 16.5±0.7 mM, RNS (Nitrate and Nitrite)- 32.0±0.95 mM, 30.9±0.63 mM, Protein carbonyl- 0.11±0.01 mg/mL as shown in Figure 1 B–E and in Table 1. A time-course study demonstrated an increase in the level of ROS-RNS-protein carbonyl in K562 cells after 1 hour of exposure to the complex. In further experiments, we showed that the complex manages to induce DNA fragmentation in K562 cells but not in NIH cells, as shown in Figure S1 H. However, the other positive dimension of the complex is its ability to reduce the cellular toxicity induced by apoptotic cells by enhancing the level of total antioxidant after 24 hours of treatment with the complex. The concentrations of SOD/TAC in the HALOA-treated cells were 4.5±0.70 mM (p=0.070) and 0.10±0.001 mM (p=0.01), and those in the untreated K562 cells were 1.0±0.0 mM (SOD) and 0.018±0.003 mM (TAC). All the data are depicted in Table 1 and presented in Figure 1 F, G.

**Table 1:** All the results indicated in the form of Mean±SD along with P-value.

S.No.	Parameters (Conc.)	Control (Mean±SD)	HLALB (Mean±SD)	OA (Mean±SD)	HALOA (Mean±SD)	P-Value
**1**.	ROS (mM)	16.5±0.7	29.0±2.82	18.0±0.0	63.5±26.1	0.044, 0.019, 0.008
**2**.	Nitrate (mM)	32.0±0.95	33.1±0.42	34.2±0.21	35.6±1.13	0.006, 0.003, 0.014
**3**.	Nitrite (mM)	30.9±0.63	34.1±0.78	35.5±0.91	36.8±0.77	0.01, 0.12, 0.01
**4**.	Protein Carbonyl (mg/mL)	0.11±0.01	0.11±0.01	0.12±0.03	0.13±0.004	0.04, 0.024, 0.0001
**5**.	TAC (mM)	0.018±0.003	0.065±0.001	0.051±0.02	0.10±0.001	0.018, 0.296, 0.01
**6**.	SOD (mM)	1.0±0.0	3.0±1.41	3.0±1.41	4.5±0.70	0.295, 0.295, 0.070
**7**.	Leptin (pg/mL)	23.1±1.36	24.4±2.47	22.1±0.51	27.8±1.36	0.046, 0.12, 0.022
**8**.	IL-8 (pg/mL)	50.9±3.96	28.5±0.57	18.4±1.13	8.08±1.09	.062, .039, .030
**9**.	MMP-9 (pg/mL)	94.2±0.77	54.8±7.63	51.13±0.02	48.0±0.77	.095, .008, .015
**10**.	Survivin (pg/mL)	2751±48.08	2652.5±62.93	2545.5±58.689	2504±29.69	.068, .023, .033

### Leukemia cells show higher expression of 18S rRNA (TaqMan)

Nuclear DNA has long been considered the primary target for bioreductive anticancer drugs [16]. The most abundant RNA species in eukaryotes is rRNA, which is required to form the ribosomal complex and produce all cellular proteins [17]. It has been noted that there is an increase in ribosome number in tumor cells compared to their normal counterparts [18]. A decrease in the expression of 18S rRNA is expected to cause deficient ribosomal assembly and/or function, genome-wide translational inhibition, and cell death [19]. We found that 18S rRNA was highly abundant in K562 cells. We compared the Ct value, as we know that the Ct level is inversely proportional to the amount of target nucleic acid in the sample. However, we found that the Ct values of the positive control (PTC) and control (K562 cells) were 15.1269±0.0733 and 10.575±0.0050, respectively, compared to HLALB- 10.575±0.0050, OA- 25.075±0.1257, and HALOA- 26.4197±0.1339. On gel electrophoresis (2.5%), we performed density analysis of the band, where the expression of 18S-rRNA in PTC and control (K562) cells was upregulated compared to HALOA-treated cells, where the expression level of 18S-rRNA was found to be downregulated significantly. The gel electrophoresis and RT-PCR results indicated (Figure S1 I, J, and Table S3) that complex interaction with 18S-rRNA leads to transcript degradation in K562 cells. The overall results indicated that 18S rRNA could be used as a biomarker during the treatment of CML.

### HALOA complex can alter the molecular proliferative markers (Leptin, IL-8, and MMP-9) and apoptotic markers (Survivin) of CML cells

In the progression of CML, the driving force is the BCR-ABL oncoprotein, which perturbs the balance of cell growth and cell death in normal hematopoietic cells and controls their malignant properties [1]. However, leptin, MMP-9, and IL-8 are crucial factors during cellular proliferation in CML. We established a correlation between these factors and examined the expression level before and after treatment with the HALOA complex. The expression levels of leptin, IL-8, and MMP-9 were 23.1±1.36 pg/mL, 50.9±3.96 pg/mL, and 94.2±0.77 pg/mL in K562 cells, and 27.8±1.36 pg/mL (p=0.022), 8.08±1.09 pg/mL (p=0.30), and 48.0±0.77 pg/mL (p=0.033) in HALOA-treated cells compared with HALOA-treated cells, respectively, as shown in Figure 2 A–C and Table 1. We found that leptin levels increased significantly, while MMP-9 and IL-8 decreased significantly in HALOA-treated cells and showed a strong pearson correlation between these factors, and the results indicated that leptin was negatively correlated with IL-8 and MMP-9, whereas IL-8 and MMP-9 were positively correlated.

**Figure 2:**
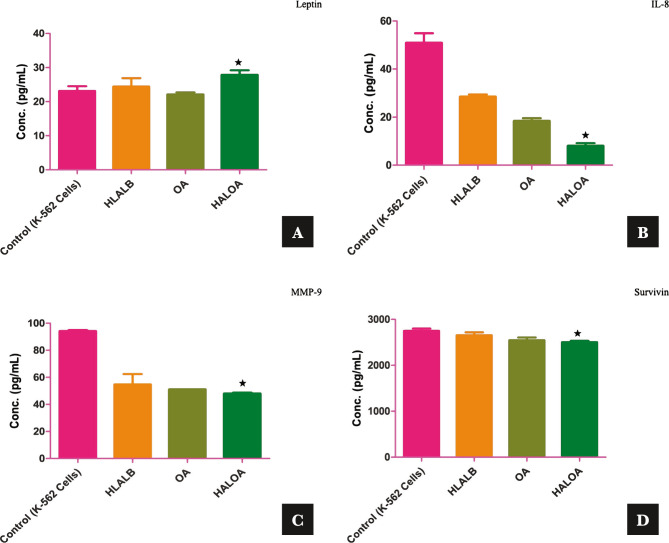
Effect of the complex on proliferative and antiapoptotic factors; (A-D) shows the conc. (pg/mL) of leptin (27.8±1.36, p-value; 0.022), IL-8 (8.08±1.09, p-value; 0.030), MMP-9 (48.0±0.77, p-value; 0.015), and survivin (2504±29.69, p-value; 0.033). (E, F) shows that the complex reduces Notch 1 and 2, which are oncogenic in nature, while it manages to upregulate the expression of Notch 4, CD-11d, and Ikaros (tumor suppressive). (G–I) docking result of the complex with Notch & RBP-Jk. The results are shown as the mean±SD. The graph shows the p-value (*<0.05), and the error bar indicates the SD. Please refer to supplementary Figure S1. Please refer to supplementary Figure S2.

**Figure 2:**
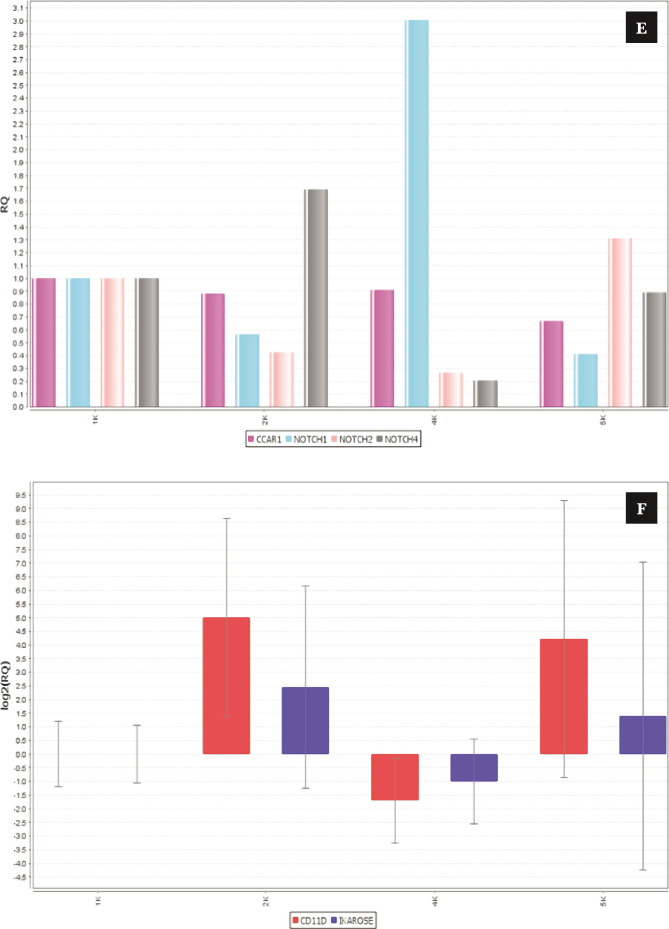
Continued.

**Figure 2:**
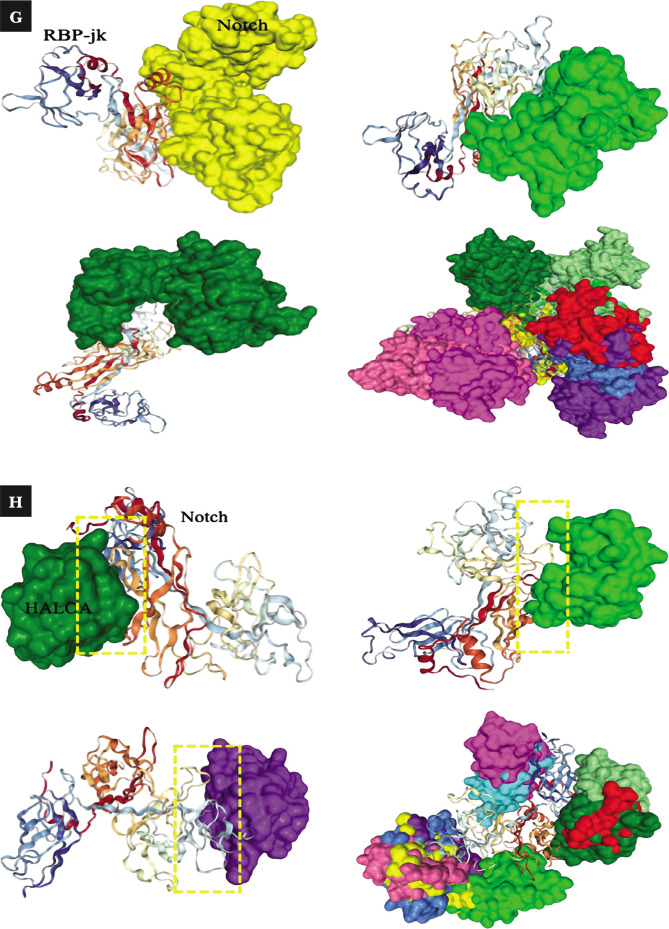
Continued.

**Figure 2:**
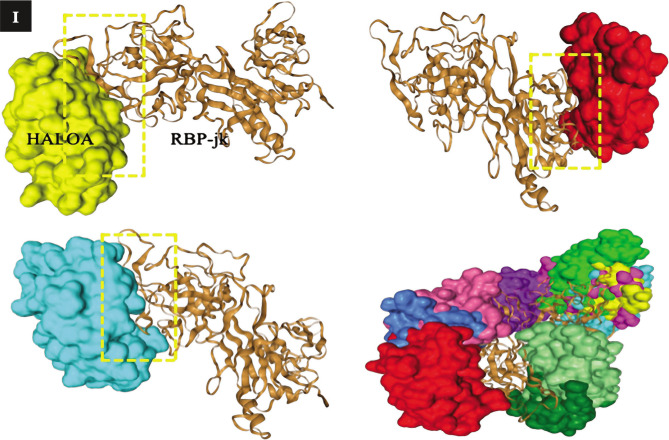
Continued.

Increased proliferation, abnormal circulation, and defects in apoptotic cell death will lead to increases in the life span of CML cells [20, 21]. In this view, regulators of apoptosis (survivin) control cell division and cell death regulation [22]. Survivin expression was found to be high in Ph-positive CML cells, while in this study, the expression level of survivin in K562 cells was 2751±48.08 pg/mL compared with that in HALOA-treated cells (2504±29.69 pg/mL, p=0.033) (Figure 2 D and Table 1), which indicates strong candidates for the complex as an antitumor agent.

### Complex targets the Notch signaling pathway (Notch-1, 2, 4, Ikaros, and CD-11d) and triggers anoikis in CML cells

Notch signaling is one of the crucial pathways that have a property in the pathogenesis of progression, while some are tumor suppressive in different kinds of cancers. In the present study, we hypothesized the targeting of notch signaling pathways by the HALOA complex in chronic myeloid leukemia (CML). Total RNA was isolated from K562 cells with and without treatment with the HALOA complex, HLALB, and OA. To confirm this hypothesis, we performed RT-PCR based on SYBR green to evaluate the expression of Notch1, 2, 4, Ikaros, and CD-11d in the control (K562 cells) and treatment groups (HALOA, HLALB, OA). All primers (Table S1) and reaction setups were performed manually. The expression of Notch 1 and 2 was found to be significantly downregulated after treatment with the complex, while Notch-4, Ikaros, and CD-11d expression was upregulated significantly (p<0.001), as shown in Figure 2 E, F (S2 A, B), and the RQ value in Table S4. The STRING result (number of nodes-11, number of edges- 12, average node degree- 2.18°, average local clustering- 0.0561, expected number of edges- 4, PPI enrichment p<0.0001) indicates that the network has significantly more interactions than expected, as shown in Figure S2 C and Table S5. Figure S2 D, E shows the ternary complex of notch/CSL/MAML, indicating that Notch can only be activated when it binds at the specific site of CSL. Figure 2 shows the binding of the Notch with RBP-Jk. Therefore, in this study, the HALOA complex blocked this binding and led to anoikis in CML cells. We validated the outcome of an *in vitro* study by the docking method, where we first analyzed the binding site of Notch and Ikaros. Both compete for the same DNA binding site at 80-87, 221-230, and 293-329 amino acid sequences on the RBP.jk protein. As the concentration of Notch was upregulated in CML, Notch showed a strong binding affinity with the RBP.jk protein at these sites, whereas Ikaros did not bind to these sites because it was downregulated in CML. However, the HALOA complex tends to inhibit the binding of the Notch on the site of the RBP.jk protein because the complex shows high binding energy to access the site efficiently in comparison to Notch, as shown in Figure 2 G–I (S2 F, G) a very strong indication of targeting with the lowest possible energy.

## DISCUSSION

Chronic myeloid leukemia (CML) is characterized by the Philadelphia (Ph) chromosome, an acquired clonal abnormality resulting from the translocation of chromosomes and the generation of BCR-ABL fusion oncogenes. In CML, inhibition of Notch1 expression in K562 cells induces erythroid maturation of leukemic cells [23]. Many previous studies have described that K562 cells are resistant to notch-1 inhibition with gamma-secretase inhibitors (GSIs) [2]. Furthermore, K562 cells show resistance to GSI in combination with either recombinant CCN-3 or Imatinib [3]. In some models, activated Notch-ICD cooperates with BCR-ABL, leading to the CML blast crisis [24]. The notch signaling pathway is involved in epithelial-mesenchymal transition (EMT). However, upregulation of the Notch pathway can reduce responses to several anticancer agents, such as cisplatin, doxorubicin, 5-fluorouracil, gemcitabine, and tamoxifen. Thus, downregulation of the notch signaling pathway appears to be a novel strategy for increasing drug sensitivity in cancer cells during conventional chemotherapies [25]. In the present study, we formulated the HALOA complex from human alpha-lactalbumin with oleic acid by direct mixing, which has the therapeutic capability to target different paradigms of chronic myeloid leukemia (CML), which are responsible for proliferation to angiogenesis. It is believed that CML cells show anchorage-independent growth and have the property to induce EMT with the help of the notch signaling pathway. Herein, we tried to examine the mechanism of action of a complex on BCR-ABL translocation, the REDOX system (ROS, RNS, protein carbonyl, TAC, and SOD), the proliferative system (Leptin, IL-8, MMP-9), the antiapoptotic system (Survivin, and CCAR1), and its effect on the Notch signaling pathway (Notch 1, 2, and 4). It has been speculated that targeting all these factors leads to anoikis in homeless CML-BC cells. The CML patients showed the two most common translocations, b2a2 and b3a2, and we also found the b3a2 major (476 bp) gene translocation in K562 cells. This study confirmed that the treatment of K562 cells with the HALOA complex led to significant inhibition of all kinds of translocations, as analyzed by density measurement (only the internal control band was visible with relative font-0.588, volume- 144970, band % – 12.2%, lane % – 11.6%) by Image Lab software. HLALB can only downregulate the translocation of b3a2 in K562 cells, but oleic acid shows no promising result in the inhibition of translocation. Therefore, the overall result indicates that the HALOA complex can inhibit BCR-ABL gene translocation. Previous literature reported that few drugs induce anoikis in BCR-ABL-positive cells by relatively enhancing the level of intracellular ROS/RNS and inducing DNA fragmentation [26].

Furthermore, our findings demonstrate that the complex manages to establish equilibrium between oxidative stress (ROS, RNS, and protein carbonyl) and antioxidant (TAC and SOD) environments, which is a favorable condition for the progression of anoikis. The HALOA complex can minimize the toxic environment induced by apoptotic cells to their surroundings during CML treatment. From this study, we are in a position to say that the HALOA complex enhances ROS/RNS/protein carbonyl, and it also elevates the production of SOD/TAC significantly (p-value of 0.01). An increase in the production of ROS/RNS/protein carbonyl leads to DNA fragmentation, which ultimately leads to anoikis in leukemia cells. Previous studies explored whether 18S-rRNA acts as an internal control during treatment with different drugs, but here, we reported that treatment with the HALOA complex downregulated 18S-rRNA, whereas 18S-rRNA was highly expressed in untreated K562 cells. EMT is the backbone for leukemia cells to become anoikis resistant, which is attained by enhancing MMP-9 and IL-8, playing an integrative role in the proliferation of leukemia cells. At the same time, leptin is also an important factor in managing normoxic conditions after the treatment process, as it helps to induce normal hematopoiesis. The results of this study also followed the same trajectory, in which the expression levels of IL-8 and MMP-9 were effectively downregulated by the HALOA complex and upregulated by the expression of leptin. We also established a protein-protein interaction within all these factors through a String web tool. Moreover, we found that the complex also decreased the antiapoptotic factors survivin and CCAR1. We can say that all the factors are somewhere directly or indirectly involved in the Notch signaling pathway; targeting the Notch signaling pathway through the HALOA complex leads to anoikis and minimizes the possible outcome of remission of leukemia after treatment. In K562 cells, Notch 1 and 2 were highly expressed, while the expression of Notch-4, Ikaros, and CD-11d was lower, but after treatment with the HALOA complex, the expression of Notch-1 and 2 was significantly downregulated, and the expression of Notch-4, Ikaros, and CD-11d was upregulated. In the case of HLALB- and OA-treated cells, we also obtained the same result, but they were not significant. An in silico study also suggested that the complex can target the Notch pathway, and the multifaceted targeting property of the HALAO complex on CML cells. Therefore, the outcome of this study itself confirms the therapeutic credential of the HALOA complex in combating CML, *i.e*., resistance against GSI and tyrosine kinase monotherapy.

## CONCLUSION

In conclusion, we can say that the pathogenesis of leukemia is not driven by single factors; rather, it is induced by combinatorial factors. These findings will help combat chronic myeloid leukemia by the HALOA complex, which can target combined factors involved in leukemogenesis.

## Supplementary Material


