# Supplementary material for: The bioengineered HALOA complex induces anoikis in chronic myeloid leukemia cells by targeting the BCR-ABL/Notch/Ikaros/Redox/Inflammation axis
Source: J Med Life. 2022 May;15(5):606–16. doi: 10.25122/jml-2021-0230 (PMC9262277; doi:10.25122/jml-2021-0230)
Supplement: Supplementary file 1 [file JMedLife-15-606-s001.pdf]

Table S1. All the primers with their sequences, annealing temperature, and amplicon length for RT-PCR.

Genes	Forward primer	Reverse Primer	Annealing Temp (°C)	Amplicon Length
**β-Actin**	TCACAATGTGGCCGAGGACTTTT	AGAAGTGGGGTGGCTTTTAGGATG	60	115
**CCAR1**	AGCTGCATTACAACAGCAATA	GGCTGTGTTAAGAGGGTTTG	60	96
**Notch 1**	AACAACAGGGAGGAGACA	GGTCCATATGATCCGTGATG	60	112
**Notch 2**	AAGGGCTTTAAAGGCTATAACT	TGTCATCAAAGCAGGTTCC	60	88
**Notch 4**	GAGGGTCCACGTTGTGAG	TCTGGGCCTATGAAACCTG	60	137
**Ikaros**	CGAATACTCTGTCTGCCCTATC	CCCATCTCTTCTCCCTCTCATA	60	122
**CD-11d (ITGAD)**	CCAGGCAATGGAGGAAGAA	CTCGGGTCTGCTCATAGTAATG	60	147

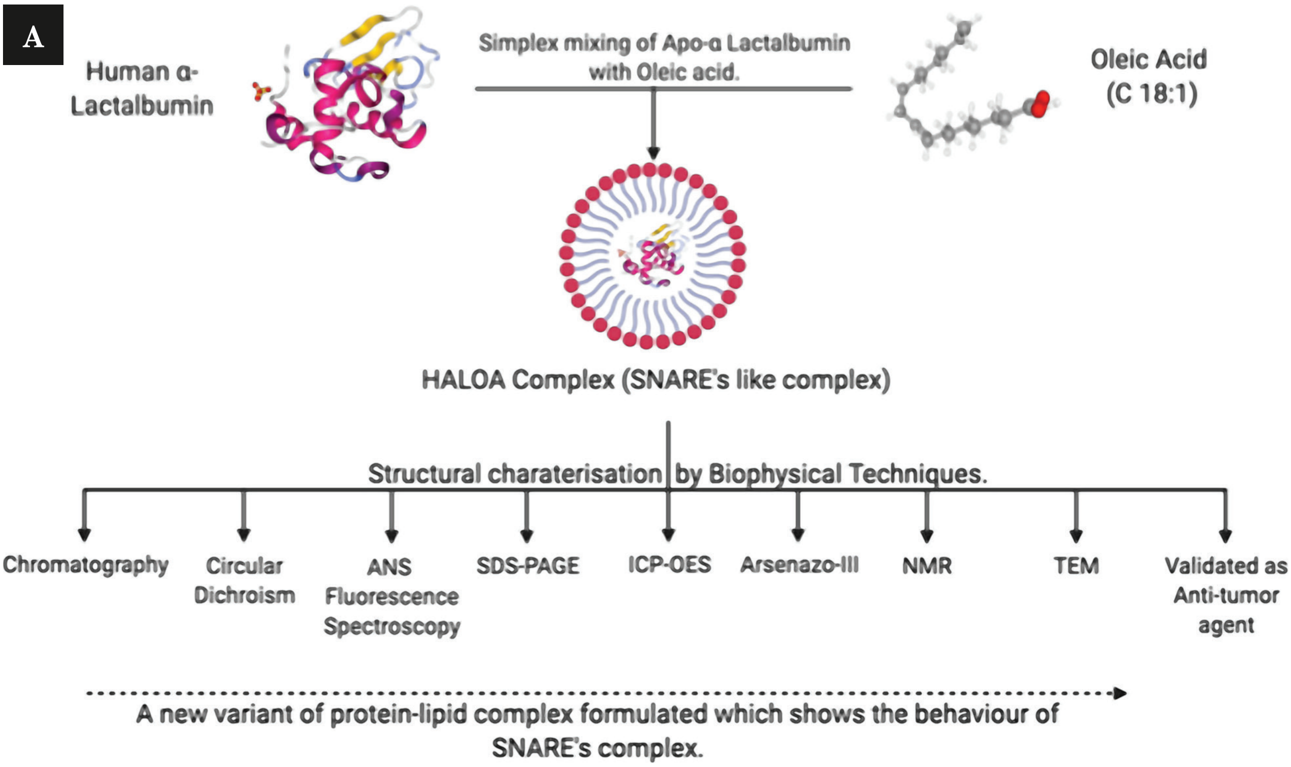

Figure S1. (A) structural characterization of the HALOA complex was previously validated by various biophysical techniques, (B) the HALOA complex led to anoikis in K562 cells but not in NIH-3T3 cells, and (C) cell viability assay after treatment with the HALOA complex at a different concentration was performed, and the IC50 (0.5 mg/mL) value was calculated throughout this study. (D–G) BCRABL1 tranlocation lane and band analysis by Image Lab software. (H) shows the DNA fragmentation result after the treamnet with complex where in the panel of HALOA complex gel image no intact DNA is shwoing, the result is analysed by image lab software. Error bar indicated the SD. (I) gel electrophoresis image clearly shows that expression of 18 s rRNA found to be high as compared to positive control while after the treatment with complex manages to downregulate the expression of 18s rRNA in k562 cells; (J) result shows the Ct value (Mean±SD) of Positive control (PTC)- 15.1269±0.0733, Control (K562)- 10.575±0.0050, HLALB- 10.2319±0.1409, OA- 25.075±0.1257, HALOA- 26.4197±0.1339, p-value; 0.001, the result indicate that lower the Ct values greater the amount of nucleic acid. The results are shown as the mean±SD. The graph shows the p-value (*<0.05), and the error bar indicates the SD.

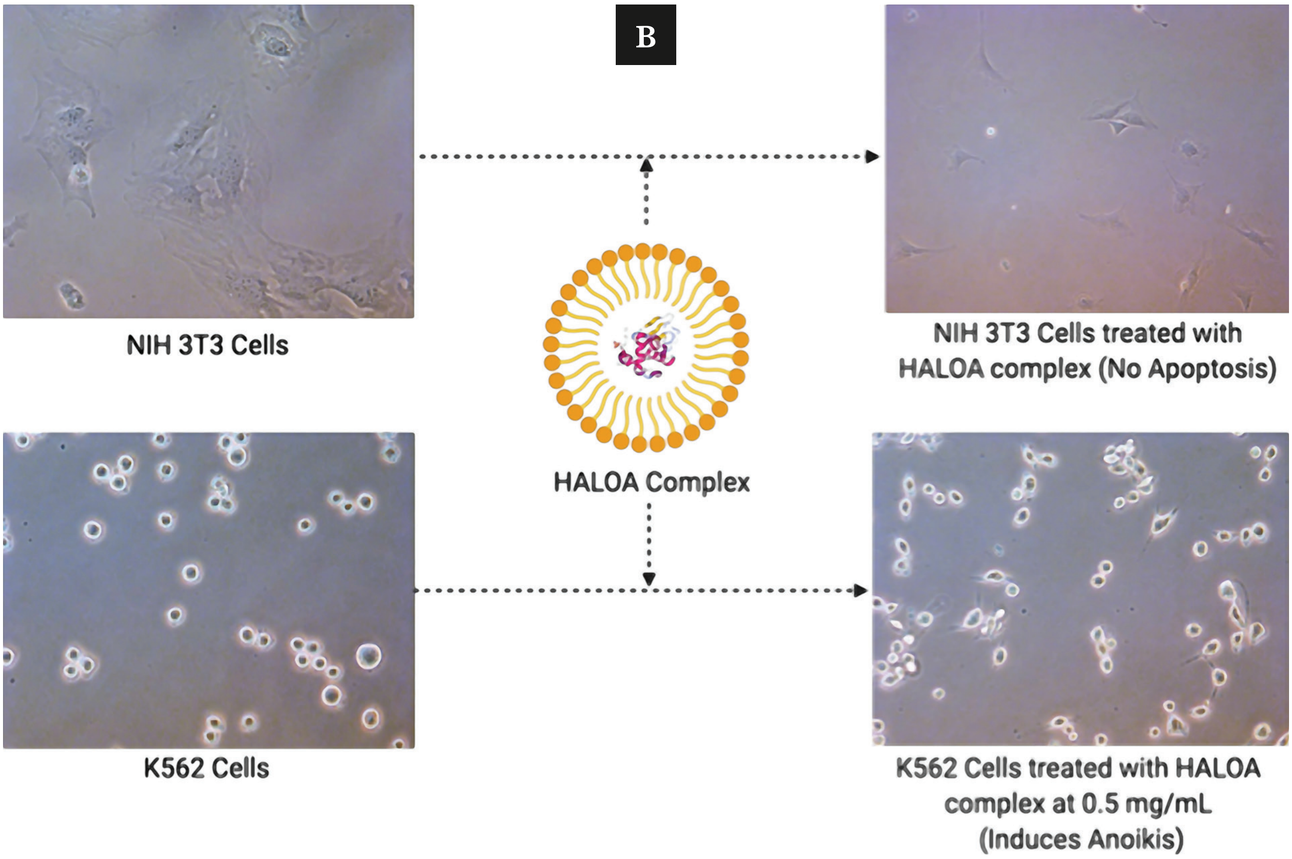

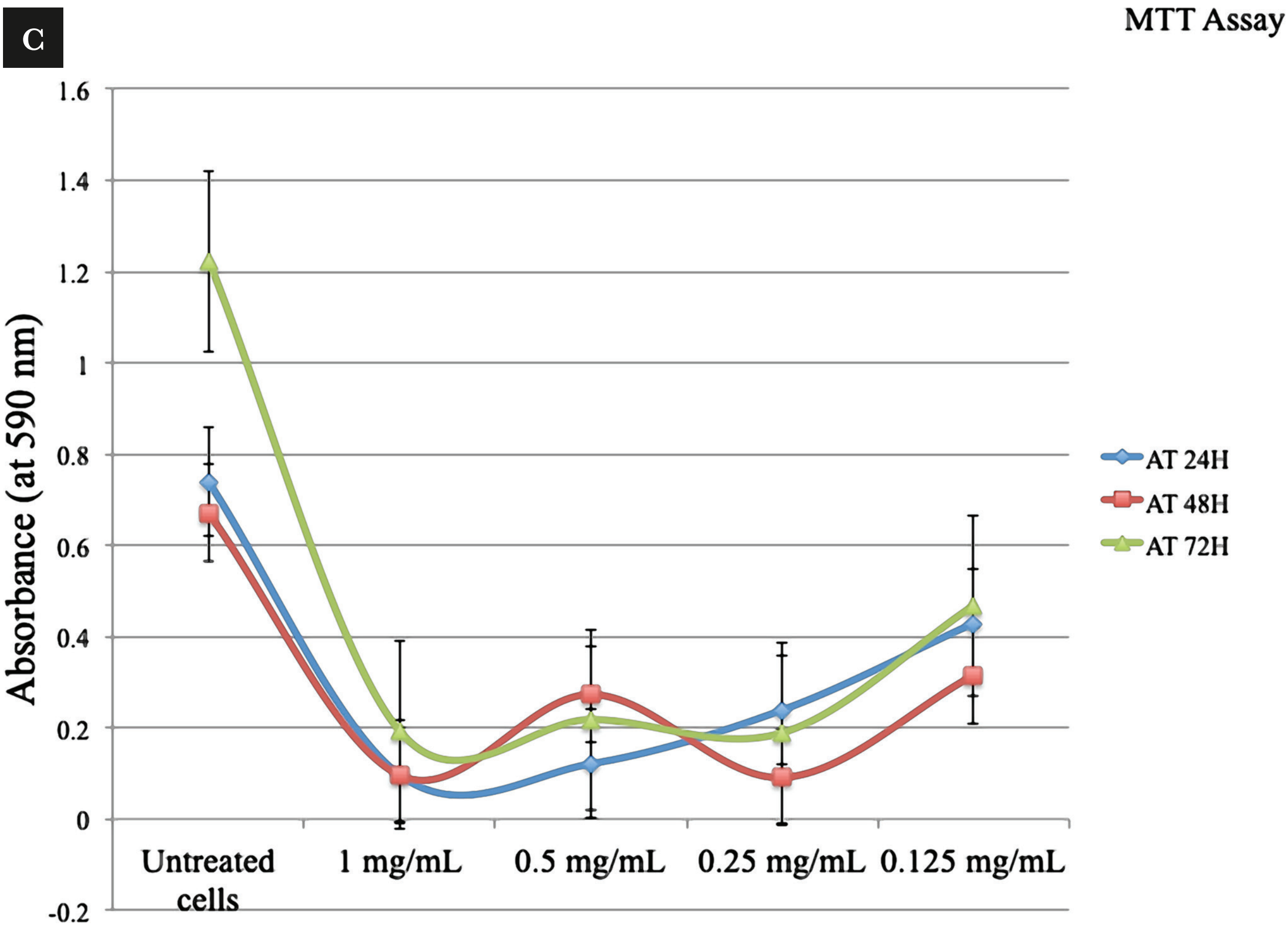

Figure S1. Continued.

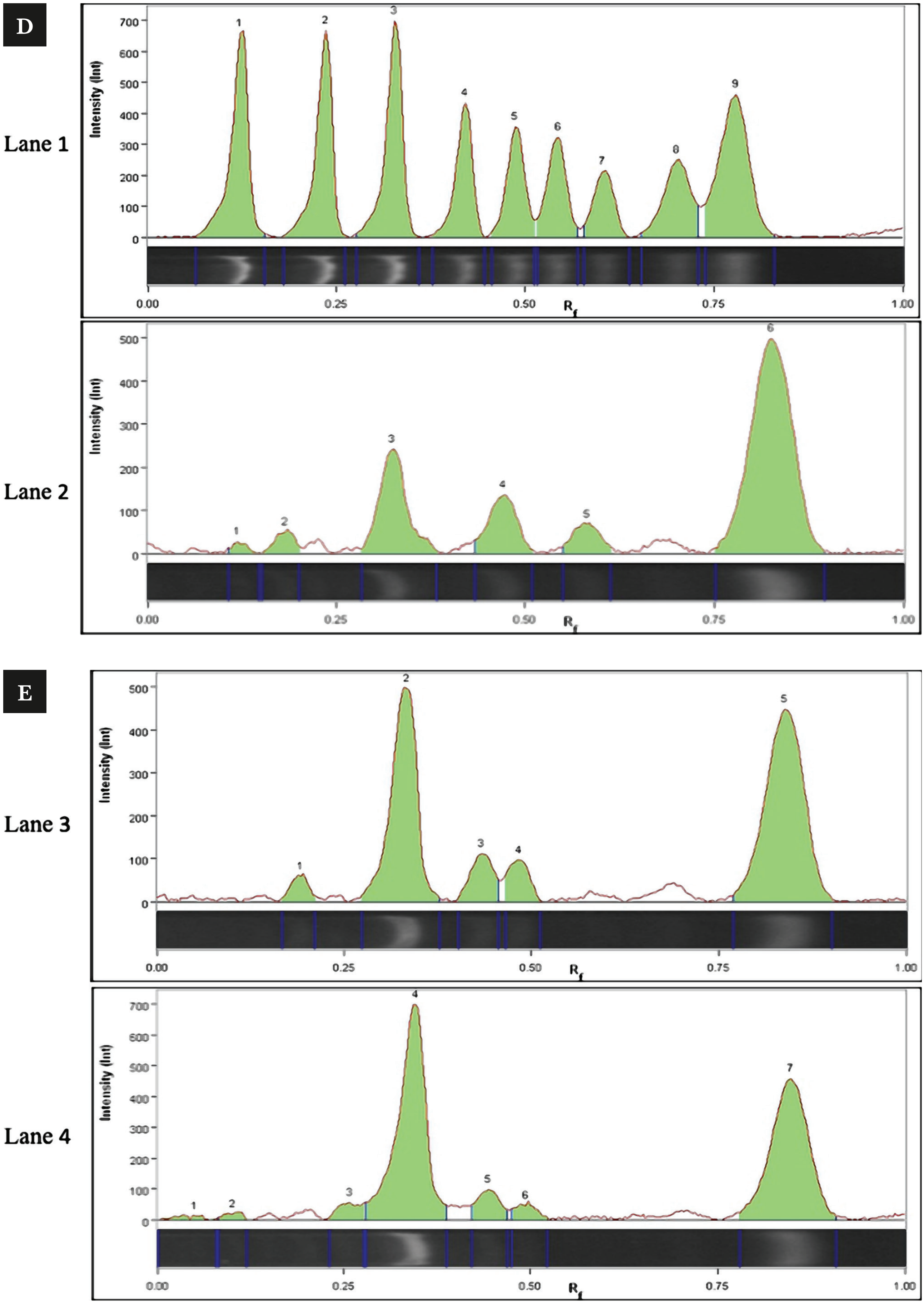

Figure S1. Continued.

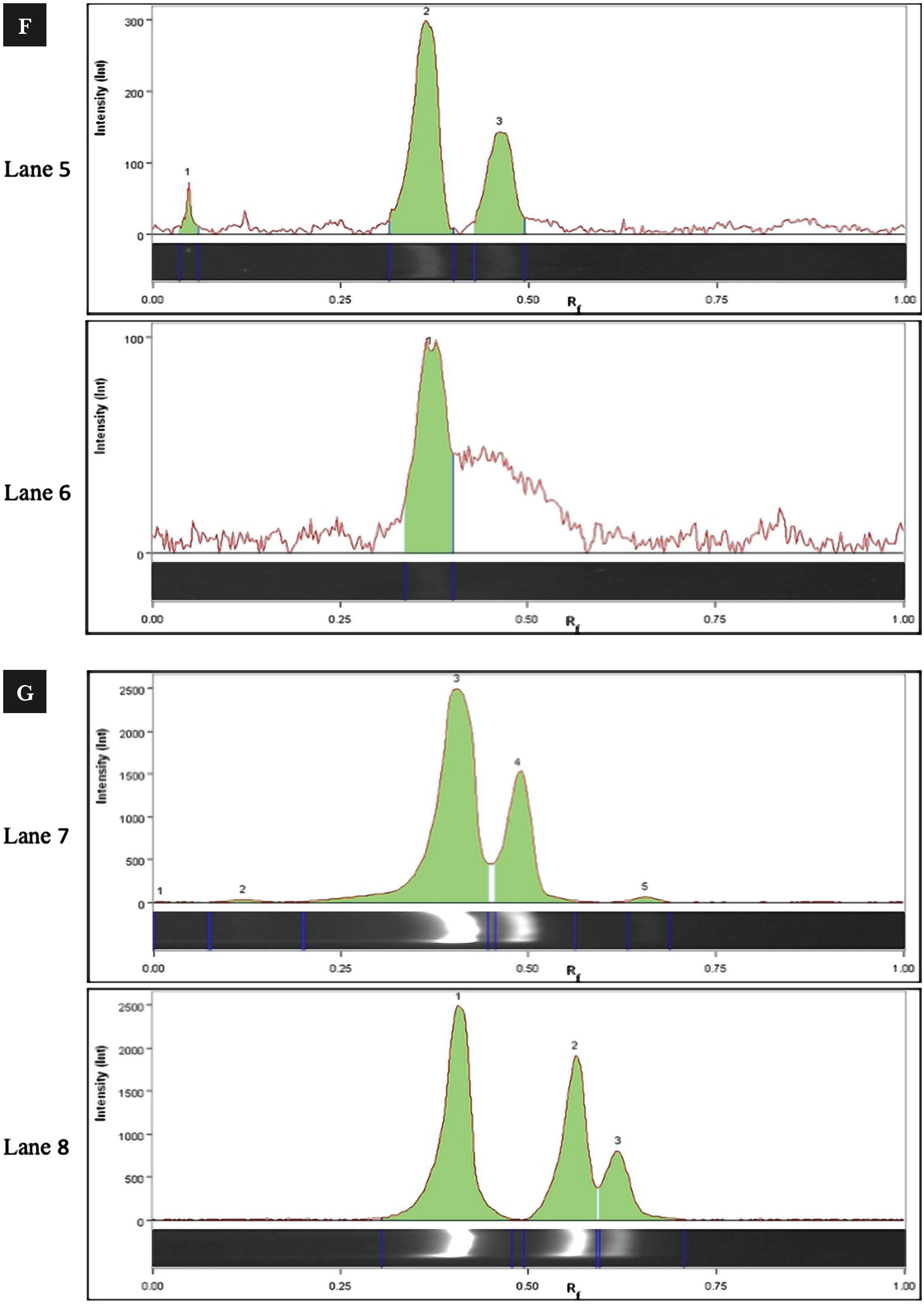

Figure S1. Continued.

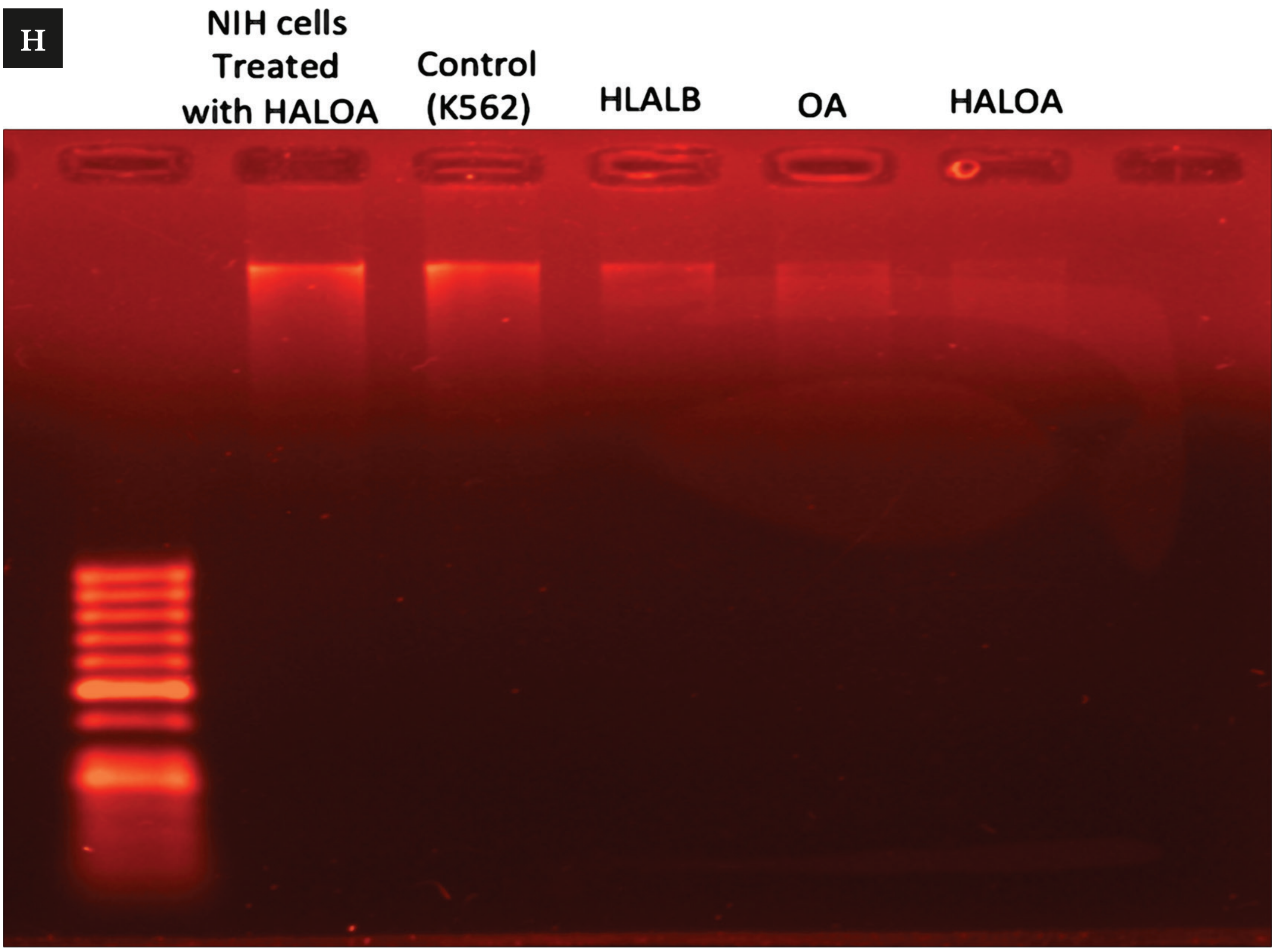

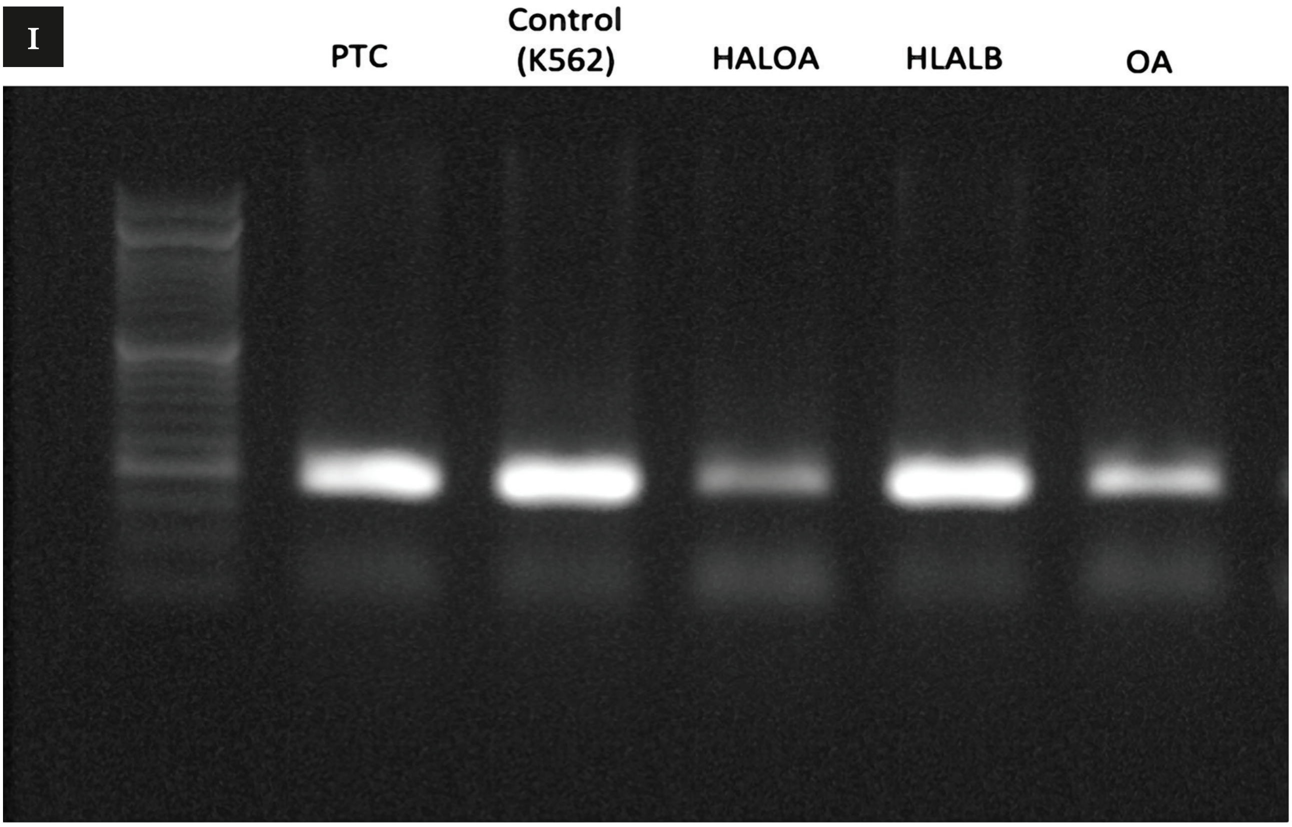

Figure S1. Continued.

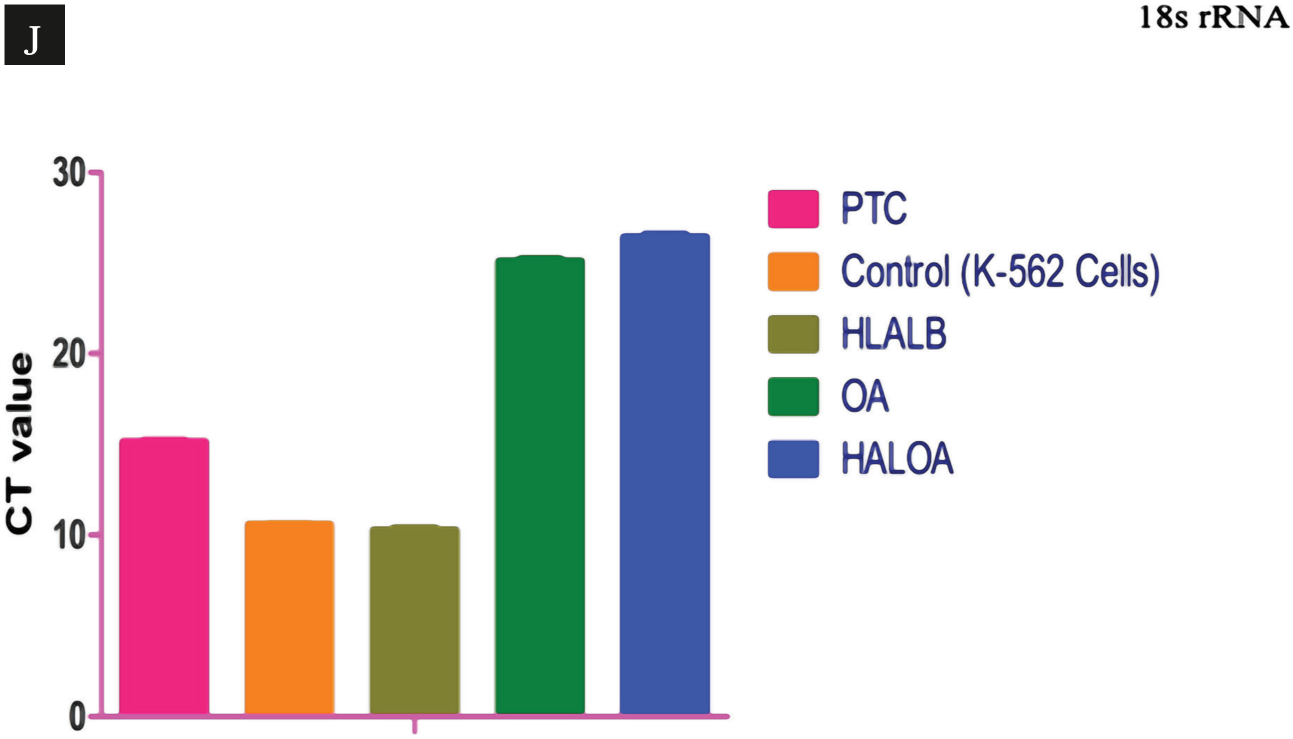

Figure S1. Continued.

Table S2. BCR-ABL translocation in all the samples (PTC-Positive control; HALOA-Human alpha-lactalbumin oleic acid complex; HLALB; Human alpha-lactalbumin; OA1, OA2, and OA3 (at different concentration).

S.No.	Name of Sample	BCR-ABL Translocation
1.	PTC	b2a2, e1a2
2.	K562 Cell line	b3a2 (major)
3.	HALOA	No BCR-ABL translocation
4.	HLALB	b3a2 (down-regulated)
5.	OA1	e1a3 and mixed
6.	OA2	e1a3 and mixed
7.	OA3	e1a3 and mixed

Table S3. Ct (Ct±SD) value of 18s rRNA of RT-PCR (TaqMan assay).

S.No.	Samples	Ct±SD
1.	Positive control (PTC)	15.1269±0.0733
2.	Control (K562)	10.575±0.0050
3.	HLALB	10.2319±0.1409
4.	OA	25.075±0.1257
5.	HALOA	26.4197±0.1339

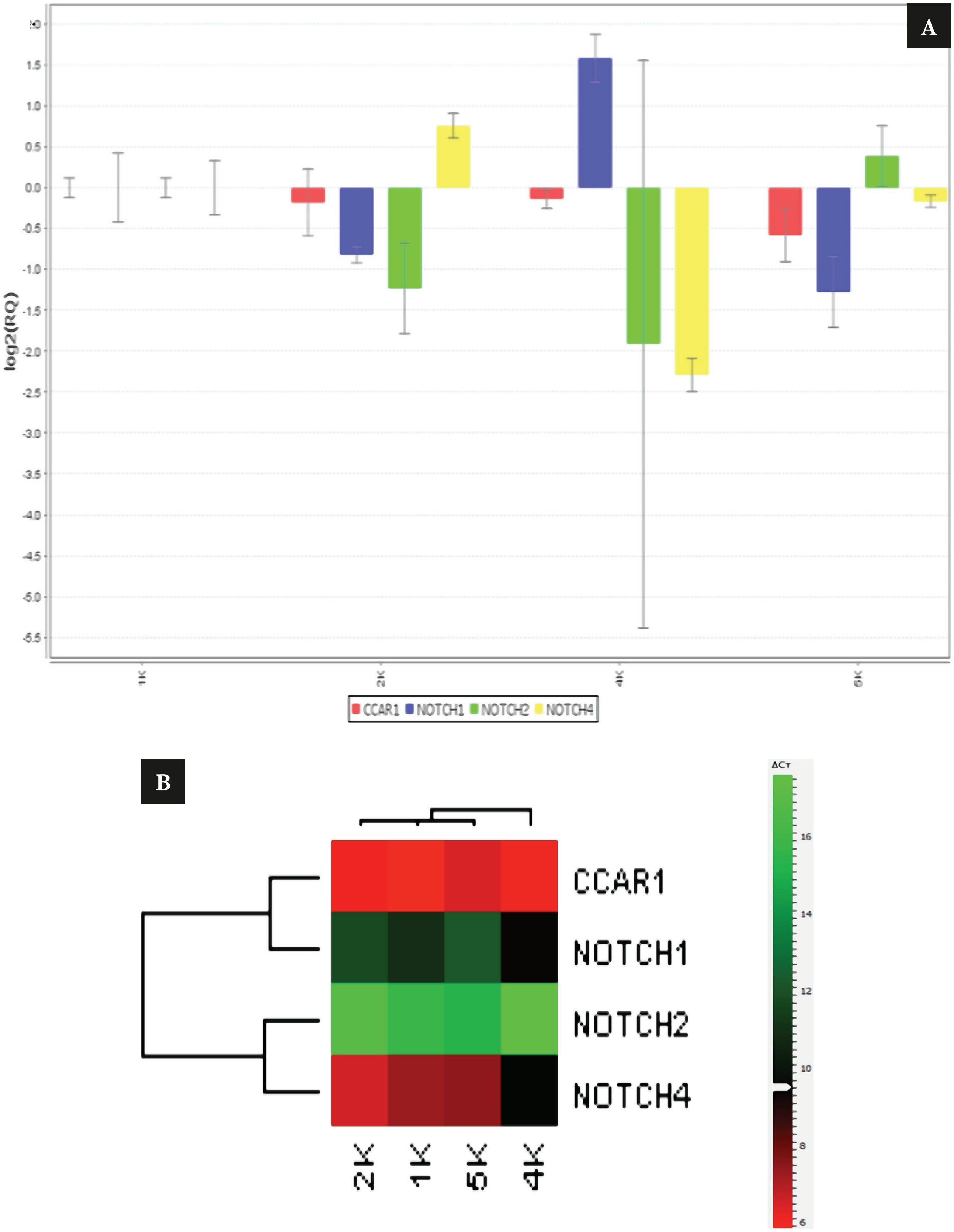

Figure S2. (A) shows that the complex reduces Notch 1 and 2, which are oncogenic in nature, while it manages to upregulate the expression of Notch 4, CD-11d, and Ikaros (tumor suppressive); (B) indicates the heat map of correlation between all these factors. The representation shows 1K-Control (K562 cells), 2K-HALOA complex, 3K-HLALB, and 4K-OA treated cells. (C) Protein-protein interactions were predicted by STRING software, which shows a strong interaction between all the factors. Error bar indicated the SD. (D) Crystal structure of activated Notch, CSL, and MAML on HES-1 promoter DNA sequence (PDB; 2F8X); (E) sequence alignment of the notch (PDB; 4J2X_1) with RBP-Jk (PDB; 2F8X_3); (F) shows the root mean square deviation (RMSD) of 1; RBP-Jk with Notch, 2; Notch with HALOA complex, 3; RBP-Jk with complex where 1-10 shows the rank of favorable docking; (G) average interaction energy score between ligand and receptors.

C

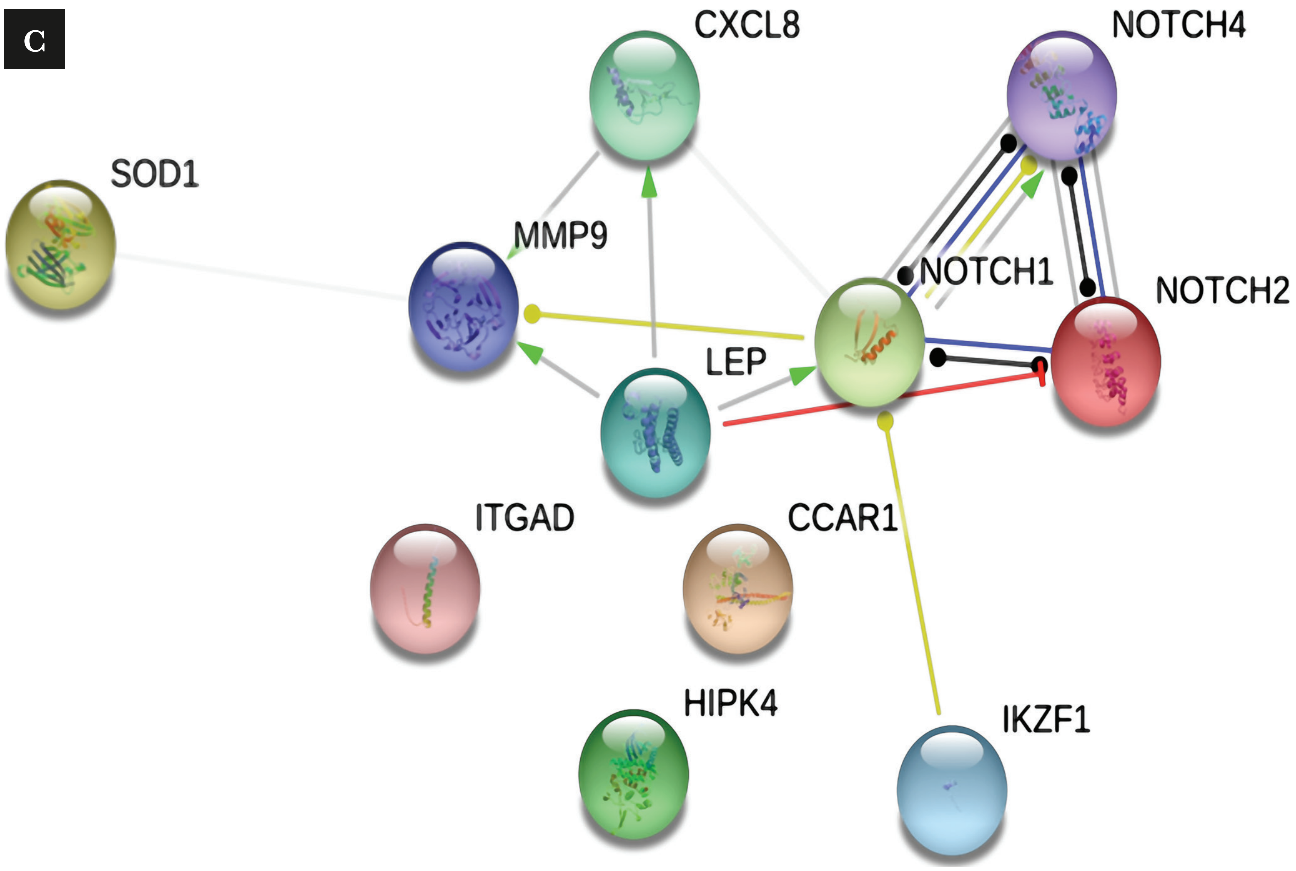

D

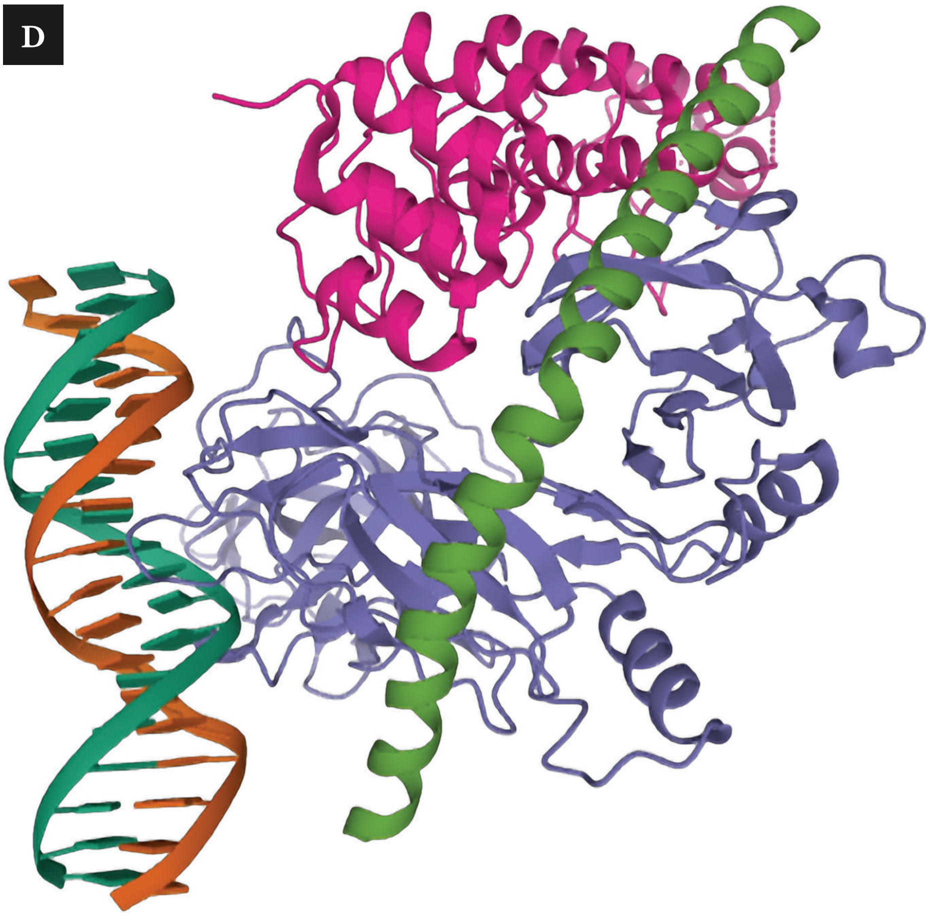

Figure S2. Continued.

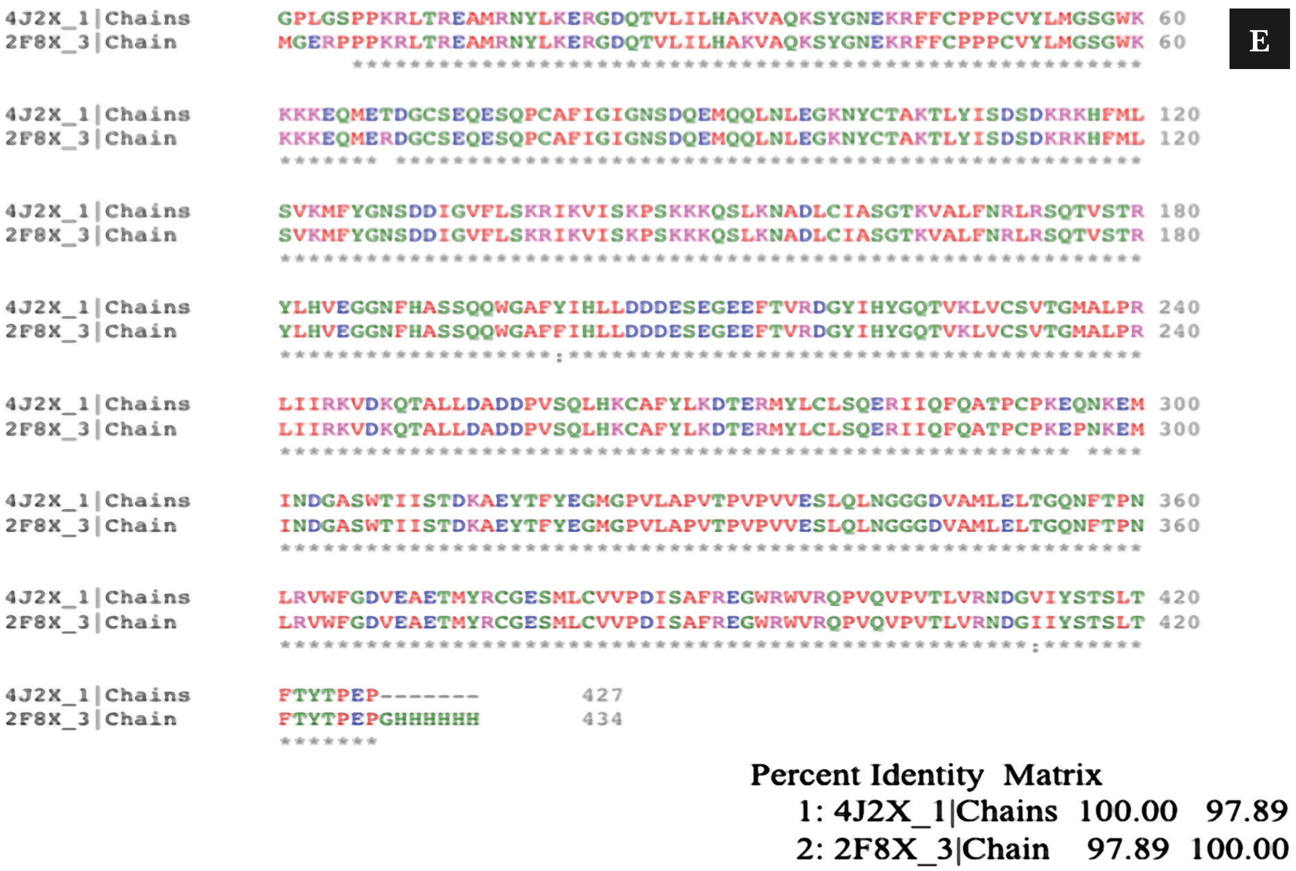

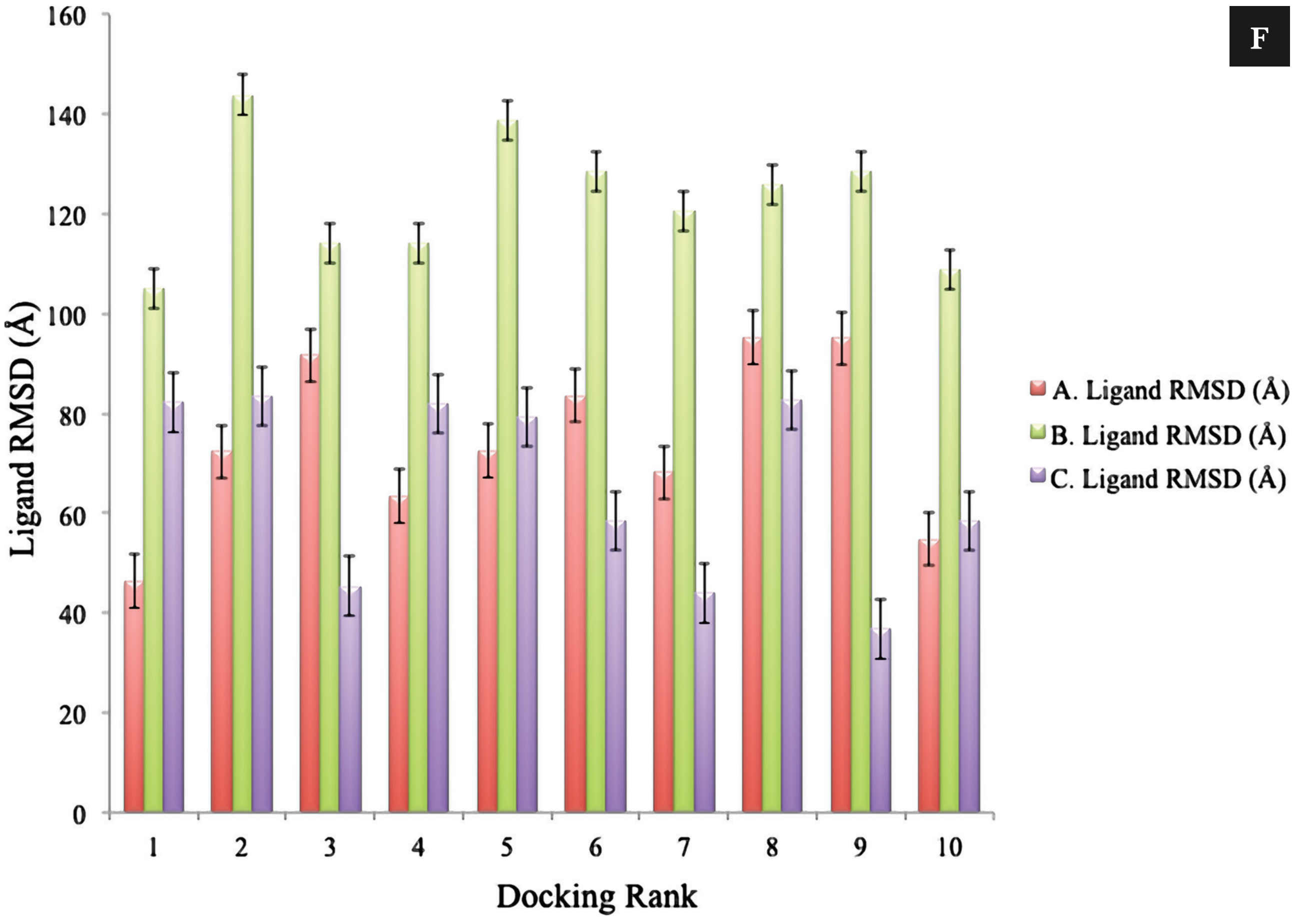

Figure S2. Continued.

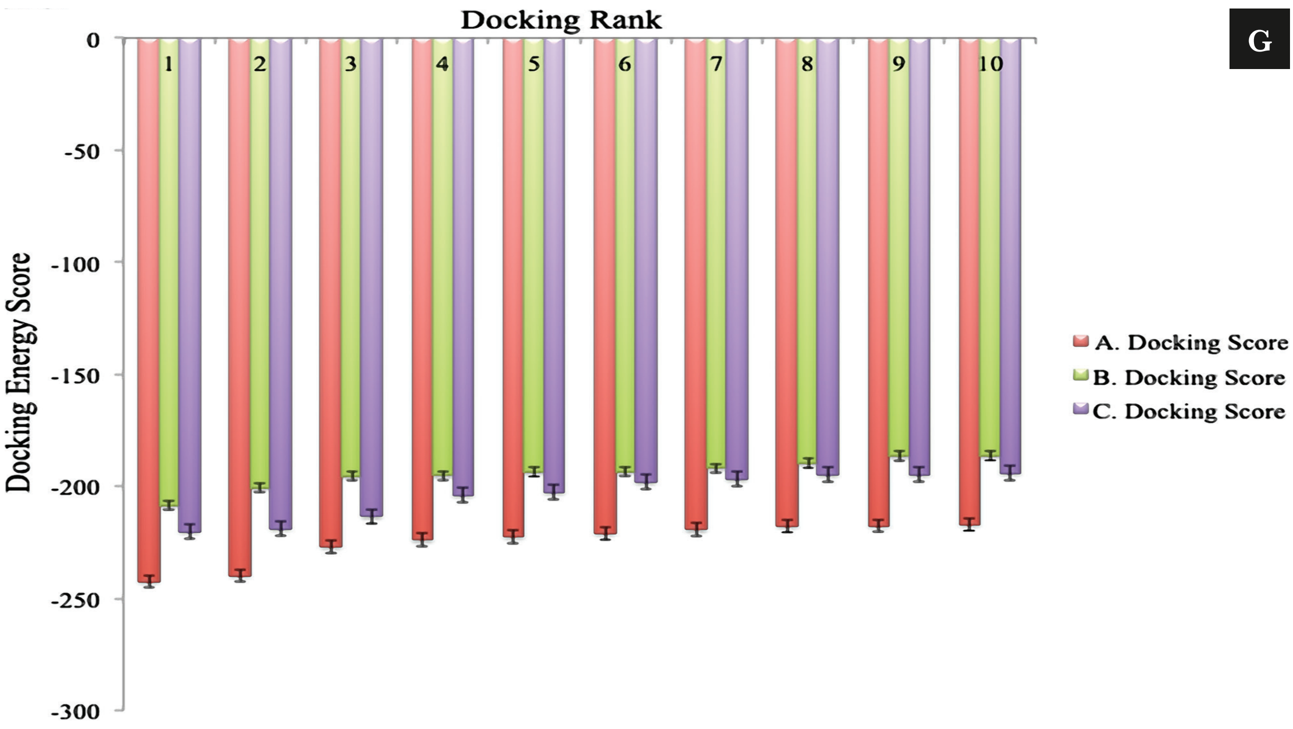

Figure S2. Continued.

Table S4. Relative fold change (RQ) results from RT-PCR analysis.

S.No.	Assay	RQ-Control (K562)	RQ-HLALB	RQ-OA	RQ-HALOA
1.	β-Actin	1	1	1	1
2.	CCAR1	1	0.9096	0.6695	0.882
3.	Notch 1	1	3.0061	0.4121	0.5655
4.	Notch 2	1	0.2662	1.3109	0.4257
5.	Notch 4	1	0.205	0.8899	1.6911
6.	Ikaros	1	0.5005	2.6418	5.4932
7.	CD-11d/ITGAD	1	0.3086	18.6956	32.2181

Table S5. Edge score of protein-protein interaction (PPI).

S.No.	Node 1	Node 2	Edge score
1.	MMP-9	CXCL8/IL-8	0.933
2.	NOTCH4	NOTCH1	0.923
3.	NOTCH4	NOTCH2	0.922
4.	NOTCH1	NOTCH2	0.913
5.	LEP	CXCL8	0.828
6.	MMP9	NOTCH1	0.764
7.	IKZF1	NOTCH1	0.746
8.	MMP9	LEP	0.707
9.	LEP	NOTCH1	0.656
10.	CXCL8	NOTCH1	0.510
11.	LEP	NOTCH2	0.450
12.	MMP9	SOD1	0.420
